# Ghrelin Restores the Disruption of the Circadian Clock in Steatotic Liver

**DOI:** 10.3390/ijms19103134

**Published:** 2018-10-12

**Authors:** Qin Wang, Yue Yin, Weizhen Zhang

**Affiliations:** 1 Key Laboratory of Molecular Cardiovascular Science, Ministry of Education, Department of Physiology and Pathophysiology, School of Basic Medical Sciences, Peking University, Beijing 100191, China; wangqin@bjmu.edu.cn; 2 Department of Surgery, University of Michigan Medical Center, Ann Arbor, MI 48109-0346, USA

**Keywords:** ghrelin, circadian rhythm, steatotic liver, mTOR, S6 (S6 ribosomal protein)

## Abstract

Obese mice demonstrate disruption of the circadian clock and feeding cycle. Circulating ghrelin, a hormone secreted mainly by gastric X/Alike cells, is significantly reduced in obese humans and animals. Here, we examined whether ghrelin improves the disruption of the circadian rhythm in steatotic hepatocytes and liver. The effects of ghrelin on hepatic circadian clock genes were studied in steatotic hepatocytes and liver of mice fed a high-fat diet (HFD) for 12 weeks. The circadian clock of cultured hepatocytes was synchronized by treatment with 100 nM dexamethasone for 1 h. Ghrelin was administrated to the cultured hepatocytes (10^−8^ M) or to mice at a dose of 11 nmol/kg/d for two weeks via a subcutaneous minipump. The mRNA and protein levels of core clock genes were analyzed. Steatosis significantly blunted the circadian pattern of clock genes such as *Bmal1*, *Clock*, and *Per* in cultured hepatocytes and liver. Treatment with ghrelin markedly restored the daily rhythm of the clock genes, with a robust oscillation between peak and trough in cultured hepatocytes isolated from obese mice. It also increased the abundance and expression amplitude of clock genes in steatotic liver, causing the peak of *Clock* to shift to the dark period and the peak of *Per2* to shift to the light period compared with the control groups. Deletion of *GHSR1a* further deteriorated the derangement of clock gene patterns in obese mice. Ghrelin significantly increased the oscillations of mTOR/S6 signaling. We demonstrate that ghrelin restored the derangement of the circadian rhythm in steatotic liver via mTOR signaling.

## 1. Introduction

Circadian rhythms influence physiological processes in living systems, ranging from the daily sleep and wake cycle to lipid metabolism and cell cycle progression [1]. Mammals have developed an endogenous circadian clock located in the suprachiasmatic nuclei that responds to the environmental light–dark cycle [2]. Similar clock oscillators have been found in peripheral tissues such as the liver [3], kidney [4], and adipose tissue [5]. Light is the most potent synchronizer for the suprachiasmatic nuclei [6], whereas feeding time and different energy status appear to be the dominant timing cues (Zeitgeber) for peripheral clocks [7,8]. At the molecular level, circadian oscillations are determined by a transcriptional autoregulatory feedback loop in which Bmal1, Clock, NPAS2, and ROR proteins act as transcriptional activators, while Per, Cry, and Rev-erb function as inhibitors to produce 24 h self-sustained rhythmic transcription of their own and target genes [9,10].

Diet-induced obesity has been demonstrated to impair molecular rhythms and circadian behavioral in mice [11]. It also alters daily rhythms in the activities of key regulators of nutrient homeostasis, including mTOR and AKT [12]. Disturbance of the circadian rhythms through feeding time changes or nutritional challenges such as high-fat diet (HFD) can lead to metabolic diseases like obesity, fatty liver, and diabetes [13,14]. Restoration of the disrupted circadian rhythms thus provides an alternative strategy for the treatment of obesity and related metabolic dysfunction.

Ghrelin, a 28-aa peptide hormone produced by X/A like cells in the gastric fundus [15], is an endogenous ligand of the growth hormone secretagogue receptor 1a (GHSR1a) [16]. The plasma level of ghrelin fluctuates diurnally, with a peak in the day and a trough at night.Its level also varies with the feeding time [17]. Circulating ghrelin may act on the circadian system as a potential feedback signal for the suprachiasmatic nucleus (SCN) [18]. Silver and coworkers have discovered that ghrelin modulates the expression of *Per1* and *Per2* in the stomac [17]. This observation indicates that ghrelin can also regulate the oscillation of the peripheral circadian rhythm [19]. Whether ghrelin can influence the circadian rhythm in liver remains unknown.

In the present study, we explored the possibility that ghrelin restores the derangement of hepatic circadian rhythm induced by HFD in steatotic liver. Administration of exogenous ghrelin significantly alters molecular clock expression, leading to a robust diurnal rhythm in steatotic hepatocytes. Deletion of *GHSR1a* renders the hepatocytes more vulnerable to the disruption of the circadian rhythm.

## 2. Results

### 2.1. Synchronization of the Circadian Rhythm of Primary Hepatocytes by Dexamethasone

**Figure 1:**
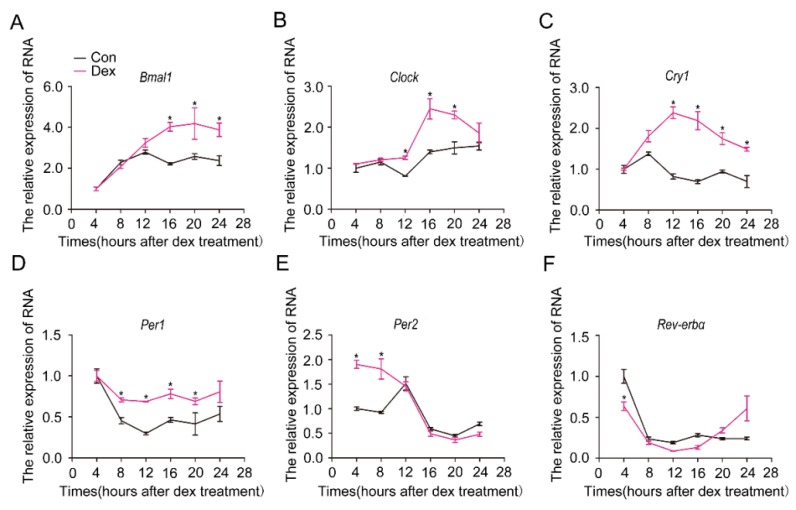
Synchronization of molecular clock genes by dexamethasone in primary hepatocytes. Hepatocytes were isolated from C57BL/6J mice and treated with 10^–7^ M dexamethasone (Dex) for 1 h. After thorough wash with PBS, fresh culture media was added. Cells were collected, and RNA was extracted at the indicated times after dexamethasone removal. Shown are the expression levels of core circadian clock genes: (**A**) *Bmal1*, (**B**) *Clock*, (**C**) *Cry1*, (**D**) *Per1*, (**E**) *Per2*, (**F**) *Rev-erbα*. Methanol was used as a control (Con, black lines). Dex (purple line): dexamethasone-synchronized group. Results were normalized to β-actin and expressed as mean ± SEM. Data are representatives of at least three separate experiments using hepatocytes isolated from distinct mice; * indicates *p* < 0.05 vs. control without Dex treatment.

Cells in vitro lose their rhythm and can be resynchronized by dexamethasone [20]. To determine whether isolated hepatocytes can be synchronized by dexamethasone, we treated the cultured hepatocytes with dexamethasone (100 nM) and analyzed the mRNA levels of circadian clock genes. As shown in Figure 1, the mRNA levels of the core circadian clock genes showed a robust rhythmic oscillation in cells treated with dexamethasone, whereas they demonstrated no obvious effect in control cells treated with methanol. We thus used 100 nM dexamethasone to synchronize hepatocytes in this study.

### 2.2. Attenuation of the Rhythm of Core Clock Genes in Steatotic Liver

**Figure 2:**
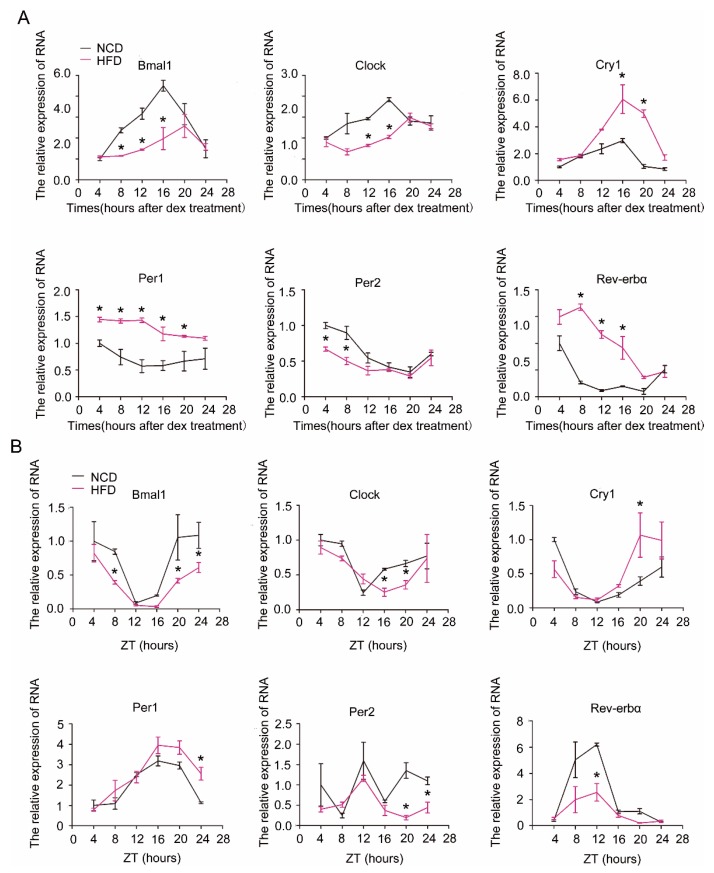
Alterations of molecular clock gene expression by high-fat diet (HFD) in primary hepatocytes and liver. (**A**) Expression of core clock genes in dexamethasone-synchronized primary hepatocytes. Primary hepatocytes were isolated from male C57B6LJ mice fed with normal chaw diet (NCD, black lines) or 60% HFD (purple lines) for 12 weeks. Shown are representatives of at least three separate experiments using hepatocytes isolated from distinct animals. (**B**) Levels of core clock genes in liver. Liver tissues were harvested every 4 h from mice fed either NCD (black lines) or HFD (purple lines) for 12 weeks. ZT = Zeitgebers time. Results were normalized to β-actin and expressed as mean ± SEM; *n =* 6–8 per group per time point; * indicates *p* < 0.05 vs. NCD mice.

We next examined the effect of HFD on the expression rhythm of clock genes in hepatocytes and liver. As shown in Figure 2, the expression amplitude or rhythmicity of the core clock genes in hepatocytes (Figure 2A) or liver (Figure 2B) was severely impaired by HFD. The amplitude and oscillation of *Bmal1* and *Clock* were significantly blunted, while *Cry1*, *Per1*, and *Rev-erbα* were increased in hepatocytes or liver of HFD mice relative to normal chaw diet (NCD) mice, resulting in attenuated diurnal variation in each of these transcripts.

### 2.3. Restoration of the Rhythm of Core Clock Genes in Steatotic Liver by Ghrelin

**Figure 3:**
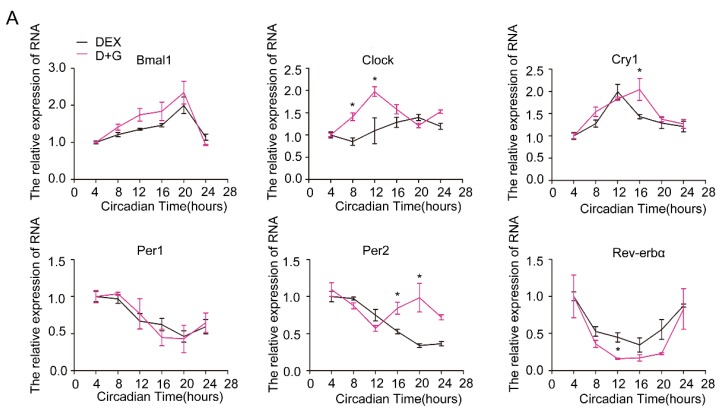

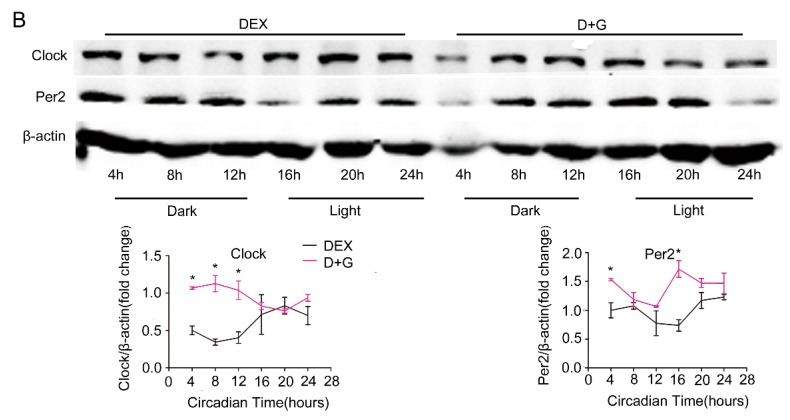
Restoration of molecular clock expression rhythm by ghrelin in primary hepatocytes. Primary hepatocytes were isolates from male C57B6LJ mice fed 60% HFD for 12 weeks. After synchronization with dexamethasone for 1 h, the cells were treated with saline (black lines) or 10^−8^ M ghrelin (D+G (Dex+Ghrelin), purple lines). Results were normalized to β-actin and expressed as mean ± SEM. (**A**) mRNA levels of core clock genes. (**B**) Protein levels of Clock and Per2. Signal intensity was normalized to that of the internal control β-actin; *n* = 9 separate experiments per group per time point; * indicates *p* < 0.05 vs. control without ghrelin treatment.

Preliminary experimental results showed that ghrelin decreases in the circulation of HFD mice. To test whether the decline of ghrelin contributes to the attenuated circadian rhythm in steatotic liver, we administrated ghrelin in vitro and in vivo. As shown in Figure 3A, treatment of hepatocytes isolated from mice fed HFD for 12 weeks with 10^−8^ M ghrelin significantly restored both amplitude and rhythmicity of the diurnal expression of *Clock* and *Per2*. The circadian expression of *Clock* was phase-advanced of 8 h, and the peak–trough amplitude was augmented in cells treated with ghrelin. On the other hand, the expression of *Per2* was phase-delayed for 12 h, and the peak of *Cry1* was phase-delayed for 4 h.

Consistent with the alteration in the mRNA levels, the protein levels of Clock and Per2 also showed a significant change after ghrelin administration (Figure 3B). The circadian expression of Clock protein was phase-advanced of 12 h, while Per2 protein was phase-delayed for 12 h. This result indicates that ghrelin enhances the phase and amplitude rhythm of clock genes in steatotic hepatocytes.

**Figure 4:**
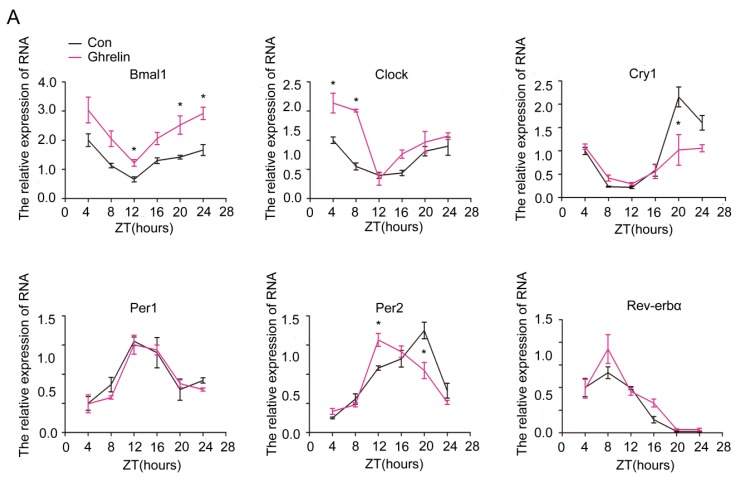

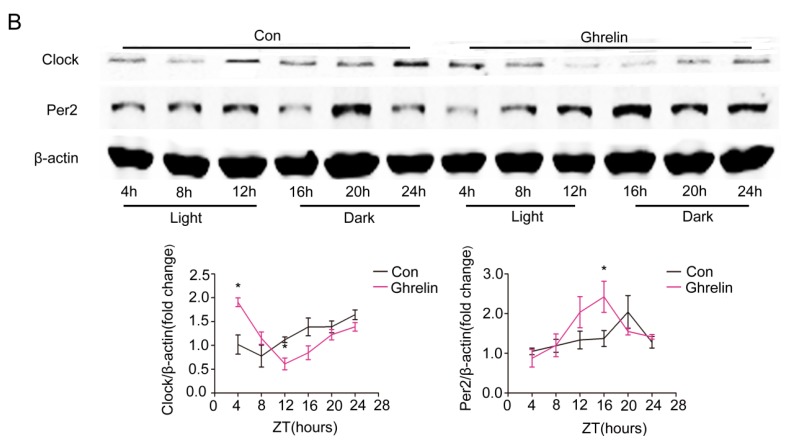
Restoration of molecular clock gene expression rhythm by ghrelin in liver. Hepatic tissues were harvested every 4 h from HFD mice that received subcutaneous administration of saline (black lines) or ghrelin (11 nmol/kg/d) (purple lines) for two weeks via a minipump. (**A**) mRNA levels of core clock genes; and (**B**) Protein levels of Clock and Per2. Signal intensity was normalized to the internal control β-actin. The results were expressed as mean ± SEM; *n =* 6–8 per group per time point; * indicates *p* < 0.05 vs. saline administration.

To further explore the effect of exogenous ghrelin on the circadian rhythm in steatotic liver, we administrated synthetic ghrelin (11 nmol/kg/d, 2 weeks) to mice fed with HFD for 12 weeks. The administration of exogenous ghrelin significantly increased the circulating levels of ghrelin (Figure S1). As shown in Figure 4, ghrelin also significantly restored the derangement of core clock gene mRNA and protein levels. Upon treatment with ghrelin, the amplitude of *Bmal1* and *Rev-erbα* mRNA was significantly increased, while both the amplitude and the rhythmicity of *Clock* and *Per2* were enhanced. The peak–trough amplitude of *Clock* was delayed for 4 h, while that of *Per2* was advanced of 8 h (Figure 4A). The protein levels of Clock and Per2 also displayed a robust diurnal variation after ghrelin administration. The peak of Clock was delayed for 4 h, while that of Per2 was advanced of 4 h (Figure 4B).

Collectively, these findings reveal that ghrelin markedly increases the expression amplitude of circadian clock genes, causing a peak shift to the dark period for *Clock* and a peak shift to the light period for *Per2* in steatotic hepatocytes and liver.

### 2.4. Effects of GHSR1a Deletion on the Circadian Clock

**Figure 5:**
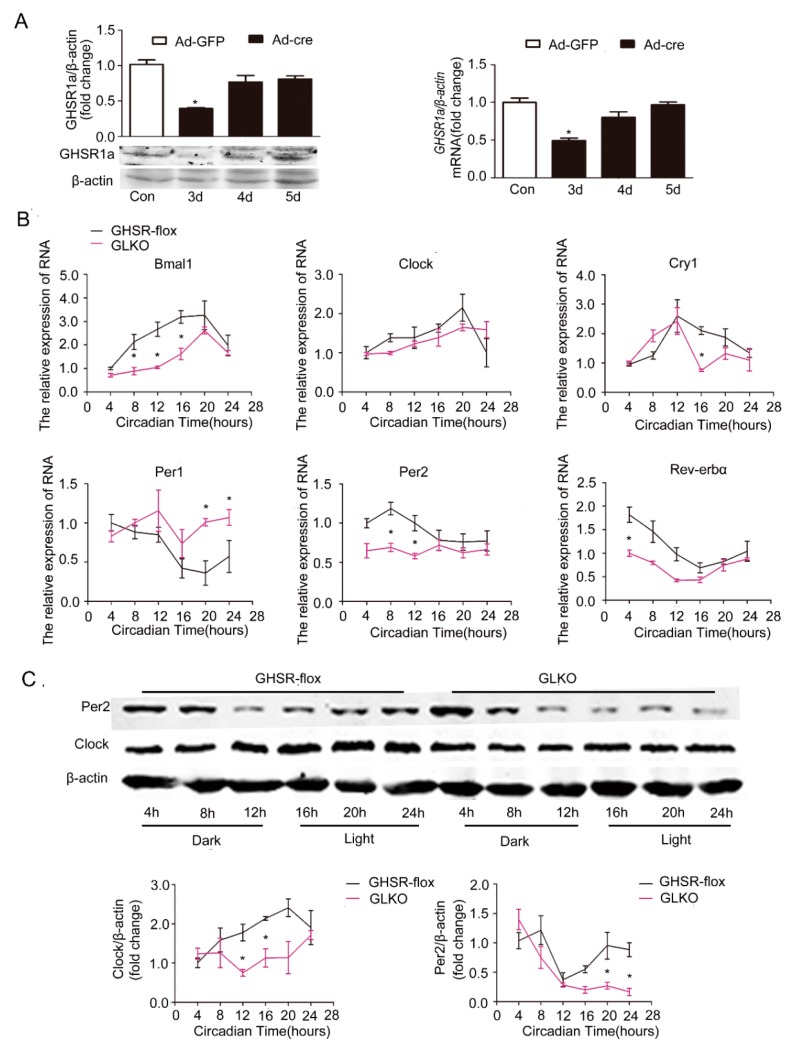
Attenuation of the circadian clock by deletion of *GHSR1a* (GLKO) in primary hepatocytes. GHSR^flox/flox^ (GHSR-flox) C57B6LJ mice (*n =* 12) were injected with GFP adenovirus (Ad-GFP) or Cre adenovirus (Ad-cre) through the tail vein for 3 days. Livers were collected, and hepatocytes were isolated and cultured for 24 h; then, proteins and RNA were extracted. The experiments were repeated for at least three separate times, using individual mice. The results were normalized to β-actin and expressed as mean ± SEM; * denotes *p* < 0.05 vs. Ad-GFP. (**A**) Validation of deficiency of GHSR1a protein expression by Western blotting and of mRNA by RT-qPCR. (**B**) mRNA levels of core clock genes. (**C**) Protein levels of Clock and Per2. The upper panel shows a representative Western blot, and the lower panel shows the quantification of the signal intensity normalized to the internal control β-actin; * indicates *p* < 0.05 vs. Ad-GFP.

We next examined the effect of endogenous ghrelin using GHSR1a-deficient mice. Cre adenovirus (Ad-cre) was injected through the tail vein into GHSR1a^flox/flox^ mice fed the HFD. The significant reduction of *GHSR1a* mRNA and protein in liver was validated by Western blotting and RT-qPCR three days after injection of Ad-cre (Figure 5A). GFP adenovirus (Ad-GFP) was used as a control. As shown in Figure 5B, deficiency of *GHSR1a* resulted in a significant decrease of the amplitude of *Bmal1*, *Clock*, *Per2*, *Rev-erbα*. Of note, GHSR1a deficiency led to an almost complete disruption of *Clock* and *Per2* mRNA oscillation (Figure 5B) in steatotic hepatocytes. Consistently, the circadian oscillation of Clock and Per2 proteins demonstrated a significant attenuation in GHSR1a-deficient mice fed with the HFD (Figure 5C). Our result suggests that endogenous ghrelin regulates the rhythm of the hepatic clock.

### 2.5. Regulation of Hepatic mTOR Activity Rhythm by Ghrelin

In summary, these findings suggest that ghrelin restored the circadian rhythm in steatotic liver likely through a mechanism dependent on mTOR/S6 signaling.

**Figure 6:**
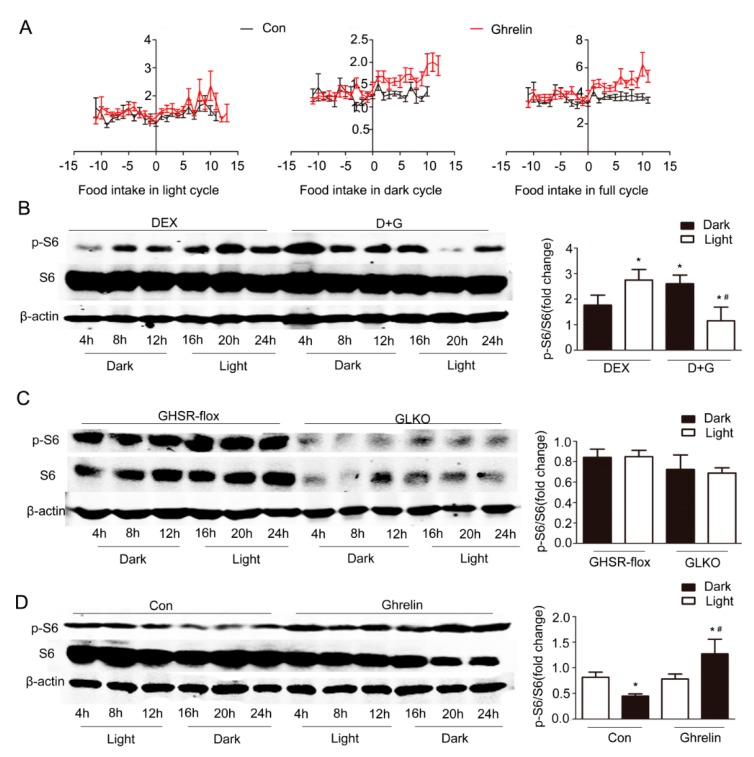
Increment of p-S6 in the dark period by ghrelin. (**A**) Food intake in light, dark, and full cycle. C57B6LJ mice fed HFD for 12 weeks were administrated saline (black lines) or ghrelin (11 nmol/kg/d) (red lines) for two weeks via a subcutaneous minipump. Day 0 was defined as the day of minipump implantation. (**B**) Effects of ghrelin on the protein levels of S6 and p-S6 in primary hepatocytes. Primary hepatocytes were isolated from male C57B6LJ mice fed 60% HFD for 12 weeks. The cells were treated with saline or 10^−8^ M ghrelin after dexamethasone synchronization. The results shown are representative of at least three individual experiments; * indicates *p* < 0.05 vs. Dex treatment dark period; ^#^ indicates *p* < 0.05 vs. D+G treatment dark period. (**C**) Effects of GHSR1a deficiency on the protein levels of S6 and p-S6 in primary hepatocytes. GHSR^flox/flox^ mice (*n =* 6 per group per time point) were injected with Ad-GFP (GHSR-flox,) or Ad-cre (GLKO,) adenovirus through the tail vein. Three days later, hepatocytes were isolated, synchronized by dexamethasone, then harvested at the indicated times. (**D**) Effects of ghrelin on the protein levels of S6 and p-S6 in liver. Tissues were harvested every 4 h from 12-weeks HFD mice that received subcutaneous administration of saline or ghrelin (11 nmol/kg/d) for two weeks via a minipump; *n =* 6–8 per group per time point. The left panel shows a representative Western blot, and the right panel shows the quantification of the signal intensity normalized to the internal control β-actin. The results were normalized to β-actin and expressed as mean ± SEM; * indicates *p* < 0.05 vs. saline treatment light period; ^#^ indicates *p* < 0.05 vs. ghrelin treatment light period.

Previous studies [21] have demonstrated that the diurnal rhythm of food intake is associated with the oscillation of mTOR activity, evidenced by increased pS6 levels during nighttime feeding. The diurnal rhythm of mTOR activity is significantly suppressed in mice fed a HFD. We thus tested whether ghrelin alters the diurnal rhythm of mTOR activity in C57BL/6J mice fed a HFD for 12 weeks. Ghrelin (11 nmol/kg/d) was administrated by a subcutaneous minipump for two weeks. Food intake was significantly increased by ghrelin treatment, especially in the dark period (Figure 6A). This change was associated with an increased oscillation in mTOR activity. Hepatic pS6 levels was significantly increased in the dark period in mice fed HFD or in cultured hepatocytes (Figure 6B,D). Conversely, deficiency of GHSR1a markedly attenuated the diurnal oscillation of mTOR activity (Figure 6C).

**Figure 7:**
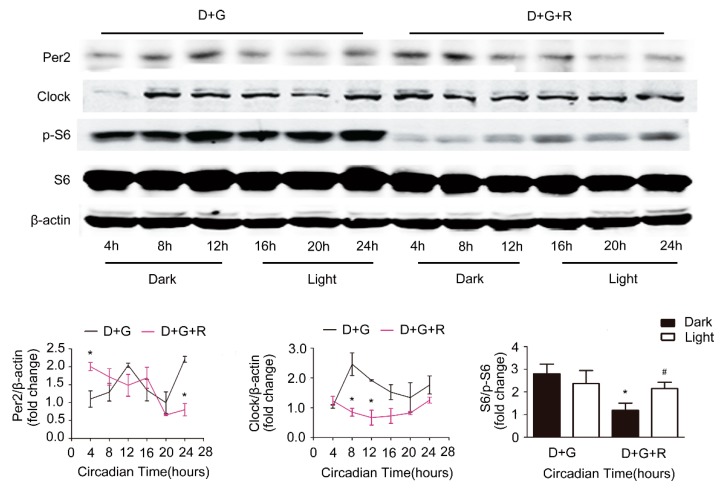
Effects of rapamycin. Primary hepatocytes were isolated from C57B6 mice fed 60% HFD for 12 weeks. Cells were synchronized with dexamethasone for 1 h, then treated with 10^−8^ M ghrelin (D+G) (black lines) or 10^−8^ M ghrelin and 20 nM rapamycin D+G+R (Dex+ghrelin+rapamycin), (purple lines). Protein levels of Clock, Per2, S6, and p-S6 were detected by Western blot. The results shown are representative of at least three individual experiments. The upper panel shows the representative Western blot, and the lower pane shows the quantification of the signal intensity normalized to the internal control β-actin. The results were normalized to β-actin and expressed as mean ± SEM; * indicates *p* < 0.05 vs. D+G treatment dark period; ^#^ indicates *p* < 0.05 vs. D+G+R treatment dark period.

To determine whether mTOR mediates the effect of ghrelin on the improvement of the circadian rhythm in steatotic liver, we used rapamycin, an inhibitor of mTOR signaling. As shown in Figure 7, rapamycin significantly decreased the protein levels of Clock, Per2, and p-S6 in cultured hepatocytes. Further, rapamycin treatment markedly perturbed ghrelin-induced restoration of the circadian rhythm, evidenced by the attenuation of diurnal variation of Clock and Per2 protein levels.

## 3. Discussion

Using pharmacological and genetic approaches, we demonstrated that ghrelin is able to restore the derangement of the circadian clock in steatotic liver by increasing the expression amplitude and shifting the expression peak of clock genes. This conclusion is supported by the following observations: (1) The amplitude or rhythmicity of the core clock genes was severely impaired in steatotic liver; (2) ghrelin increased the expression amplitude of circadian clock genes, shifting the peak of *Clock* and *Per2* to the dark period and to the light period, respectively; (3) Deletion of *GHSR1a* significantly decreased the oscillation of *Clock* and *Per2*; (4) Ghrelin enhanced the oscillation of mTOR activity, whereas deficiency of *GHSR1a* demonstrated the opposite effect; (5) Inhibition of mTOR activity attenuated the beneficial effect of ghrelin on the diurnal oscillation of core clock genes in steatotic liver.

Nutrient homeostasis is critical for the coordination of daily rhythms of activity, feeding behavior, energy utilization, and energy storage across the daily 24 h light–dark cycle. Our observation that HFD alters the period and level of the central clock in hepatocytes under conditions of ad lib access to food indicates that changes in energy homeostasis affect the molecular machinery of the clock in liver either directly or indirectly. Consistently, previous studies have shown that mice fed with HFD show a significant change in the amplitude or rhythmicity of genes involved in the circadian clock, such as *Bmal1*, *Clock*, *Per2*, and in lipid metabolism, such as *SREBP1C*, *ACC*, *PPARγ*, in liver [11].

Taken together, the previous and present findings suggest that diet-induced obesity per se might lead to altered circadian behavioral and molecular rhythms in liver. Conversely, ill-timed lifestyle patterns such as high-fat food intake, light pollution, shift work, or chronic jet lag, which significantly disrupt the circadian rhythm, may subsequently increase susceptibility to certain diseases like obesity and diabetes. Restoration of the diurnal oscillation of circadian clock genes may thus hold promise for the treatment of obesity and metabolic diseases.

Our studies reveal a novel function of ghrelin in the regulation of hepatic clock rhythm. Consistently, previous studies have shown that ghrelin directly acts on the SCN to induce phase advances in the circadian rhythm [18]. Further, ghrelin has been reported to regulate the expression of Per1 and Per2 in the stomach [17]. All these observations indicate that ghrelin plays an important role in the modulation of circadian rhythm, acting either on the SCN or on peripheral tissues. The physiological significance of hepatic clock control by ghrelin remains unknown. Our previous study demonstrated a direct action of ghrelin on hepatocytes to regulate de novo lipogenesis [22], suggesting the presence of a functional GHSR1a in hepatocytes. Since hepatic lipogenesis undergoes a diurnal oscillation, we would propose that ghrelin acts on hepatocyte GHSR1a to alter clock genes expression, leading to the diurnal variation of triglyceride synthesis in the liver. Supplementation of exogenous ghrelin, which is significantly reduced in HFD-induced obesity, may restore the oscillation of hepatic clock genes and the dysfunction of lipid metabolism in the liver. In line with this concept, the administration of ghrelin has been shown to increase locomotor activity and subsequent diurnal pattern in food anticipatory behavior [17], while the absence of GHSR1a diminished this pattern [23]. In agreement with previous reports showing levels of circulating ghrelin corresponding to 100–110 pg/mL in obese subjects [24], we also detected a significant decrease in plasma ghrelin (100–120 pg/mL) in mice fed HFD. Supplementation of exogenous ghrelin increased its level to values comparable to those of mice fed NCD (150–170 pg/mL).

mTOR signaling plays an essential role in energy homeostasis [25]. Interestingly, our studies show that the diurnal oscillation of mTOR activity was dramatically reduced in steatotic liver. Ghrelin administration restored the HFD-induced attenuation of the diurnal rhythm of hepatic mTOR signaling. Deletion of GHSR1a further impaired the reduction of mTOR oscillation in steatotic liver. Inhibition of mTOR signaling blocked the effect of ghrelin on hepatic clock genes. All these observations indicate that mTOR signaling may mediate the effect of ghrelin on the hepatic clock rhythm.

In conclusion, our study demonstrates that ghrelin could restore the circadian rhythm disrupted by HFD in the liver. Forcurrent ill-timed lifestyle patterns, ghrelin may be beneficial for liver health.

## 4. Materials and Methods

### 4.1. Materials

The ghrelin peptide was purchased from Phoenix Pharmaceuticals, Inc. (Burlingame, CA, USA). Dexamethasone was purchased from Sigma-Aldrich (St. Louis, MO, USA). Mouse anti-β-actin and rabbit anti-S6 and anti-p-S6 were obtained from Cell Signaling Technology (Beverly, MA, USA). Rabbit anti-GHSR1a and rabbit anti-Clock were purchased from Santa Cruz Inc. (Santa Cruz, CA, USA). Rabbit anti-Per2 was from MBL Inc. (Medical & Biological Laboratories, Nagoya, Japan).

### 4.2. Animals and Treatment

Male C57BL/6J mice were used in the present study. Four-week-old mice were assigned to receive a normal chow diet or a high-fat diet (60% fat, D12492; Research Diets, New Brunswick, NJ, USA) ad libitum for 12 weeks. Mice were handled in accordance with the Guide for the Care and Use of Laboratory Animals published by the US National Institutes of Health (NIH publication no. 85 revised 1996). All experimental protocols were approved by the Animal Care and Use Committee of Peking University (Permit Number: LA2012-60, 6 January 2012). All mice had free access to food and drinking water and were maintained under conditions of controlled temperature (24 °C) and humidity, with a 12 h light (lights on at 7:30 a.m.) and 12 h dark (lights off at 7:30 p.m.) cycle. For circadian studies, the animals were sacrificed every 4 h starting at 7:30 a.m. (ZT4) for 24 h (*n =* 7–8 at each time point). 

Surgery and implantation of osmotic minipumps: Mice were anesthetized with 1% Nembutal (7 mL/g body weight). Through a 1 cm incision in the back skin, the mice were implanted subcutaneously with an Alzet osmotic minipump (model 1002) filled with vehicle or acyl-ghrelin (11 nmol/kg/d) for 14 days. Before implantation, the pumps were filled with the test agent and placed in a Petri dish with sterile 0.9% saline at 37 °C for at least 4 h to prime the minipumps.

### 4.3. Isolation and Synchronization of Hepatocytes

Hepatocytes were isolated from mouse liver as previously described [26]. Briefly, male C57BL/6 mice fed with HFD were anesthetized with 1% Nembutal (7 mL/g body weight) and injected intraperitoneally with 1000 IU heparin. The liver was perfused with 20 mL pre-warmed 37 °C DHANKS buffer, followed by 15–20 mL of 3.3% collagenase IV (Sigma Aldrich Corp., St. Louis, MO, USA) at a flow rate of 4 mL/min. After perfusion, liver tissues were removed and washed with 15 mL high-glucose DMEM. The hepatocytes were centrifuged at 50 g for 3 min and washed thrice with DMEM medium to remove tissue dissociation enzymes, damaged cells, and non-parenchymal cells. Dexamethasone (100 nM) was applied for 1 h to synchronize the cells. After thorough washing, fresh culture media was added, and the cells were collected at the indicated times after dexamethasone removal.

### 4.4. Western Blotting

Cells or liver tissue were homogenized with cell lysis buffer to obtain protein lysates. Proteins were separated by SDS-polyacrylamide gel electrophoresis and transferred to polyvinylidene fluoride membranes. The membranes were incubated for 1 h at room temperature with 5% fat-free milk in Tris-buffered saline containing Tween 20, followed by incubation overnight at 4 °C with primary antibodies. β-actin was used as an internal control.

### 4.5. Analysis of Gene Expression

Total RNA was isolated with RNATrip (Applied Gene, Beijing, China) from hepatocytes or liver tissues according to the manufacturer’s protocol and reverse-transcribed into cDNAs by reverse transcriptase PCR (Cat#3500, Promega, Fitchburg, WI, USA). The cDNA was detected by the Agilent AriaMx real-time PCR system (Agilent Technologies, Inc., Santa Clara, CA, USA).

Sequences of primers for circadian rhythm genes and β-actin are shown in Table 1.

### 4.6. Statistical Analysis

Statistical analysis was performed using Graph Pad Prism Version 5.0 (GraphPad Software, Inc., San Diego, CA, USA). The differences between groups were assessed by an unpaired two-sample *t*-test, and multiple comparisons between more than two groups were analyzed by one-way ANOVA. The data represents means ± standard error of the mean (SEM). A *p*-value < 0.05 was considered significant.

## Figures and Tables

**Table 1:** Primer sequences used in qRT-PCR.

Gene	Forward (5′-3′)	Reverse (5′-3′)
*β-actin*	ATCTGGCACCACACCTTC	AGCCAGGTCCAGACGCA
*Bmal1*	TCAAGACGACATAGGACACCT	GGACATTGGCTAAAACAACAGTG
*Clock*	CAC TCT CAC AGC CCC ACT GTA	CCC CAC AAG CTA CAG GAG CAG
*Cry1*	CACTGGTTCCGAAAGGGACTC	CTGAAGCAAAAATCGCCACCT
*Per1*	CCAGCGTGTCATGATGACATAC	CTCTCCCGGTCTTGCTTCAG
*Per2*	TGTGCGATGATGATTCGTGA	GGTGAAGGTACGTTTGGTTTGC
*Rev-erbα*	TCTCTCCGTTGGCATGTCTAGA	GCAAGCATCCGTTGCTTCTC

## Supplementary Materials

The following are available online at http://www.mdpi.com/1422-0067/19/10/3134/s1.

## Abbreviations

SCN	Suprachiasmatic nucleus
ZT	Zeitgeber time
CT	Circadian time
CLOCK	Circadian Locomotor Output Cycles Kaput ear dichroism
BMAL1	Brain and muscle ARNT-like protein 1
PER	Period
CRY	Cryptochromes
REV-ERBα	Nuclear receptor subfamily 1, group D, member 1
GHS-R1α	Growth hormone secretagogue receptor 1
mTOR	Mechanistic target of rapamycin
NCD	Normal chaw diet
HFD	High-fat diet
S6	S6 ribosomal protein
