# Supplementary material for: Ghrelin Restores the Disruption of the Circadian Clock in Steatotic Liver
Source: Int J Mol Sci. 2018 Oct 12;19(10):3134. doi: 10.3390/ijms19103134 (PMC6213951; doi:10.3390/ijms19103134)
Supplement: Supplementary file 1 [file ijms-19-03134-s001.docx]

Supplemental figure1 Plasma ghrelin.


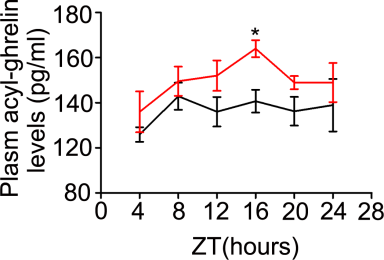
.

Plasma levels of ghrelin were measured using the ELISA assay kit from Phoenix Pharmaceuticals, Inc.(Burlingame, CA). Mice fed HFD were administrated with saline (Black lines) or ghrelin (11 nmol/kg/d) (Red lines) for 2 weeks via a subcutaneous mini-pump. Results were expressed as mean±SEM. (n=6 per group per time point). * indicates P<0.05 vs. NCD.
